# Age-related differences in borderline personality disorder traits and childhood maltreatment: a cross-sectional study

**DOI:** 10.3389/fpsyt.2025.1454328

**Published:** 2025-01-22

**Authors:** GuoRong Li, Yong Lin, Yun Xu, Yong Zhou, YanYan Wei, LiHua Xu, XiaoChen Tang, Zixuan Wang, Qiang Hu, JiJun Wang, HaiSu Wu, ZhengHui Yi, TianHong Zhang

**Affiliations:** ^1^ Department of Psychiatry, Kangci Hospital of Jiaxing, Tongxiang, Zhejiang, China; ^2^ Shanghai Key Laboratory of Psychotic Disorders, Shanghai Mental Health Center, Shanghai Jiaotong University School of Medicine, Shanghai Engineering Research Center of Intelligent Psychological Evaluation and Intervention, Shanghai, China; ^3^ Department of Clinical Psychology, Shanghai Xinlianxin Psychological Counseling Center, Shanghai, China; ^4^ Department of Psychiatry, ZhenJiang Mental Health Center, Zhenjiang, China

**Keywords:** personality disorder, borderline trait, self-report, childhood abuse, emotional abuse

## Abstract

**Introduction:**

This study investigates age-related differences in Borderline Personality Disorder (BPD) traits and childhood maltreatment (CM) experiences among adolescents, young adults, and older adults within a clinical sample.

**Methods:**

A cross-sectional design was employed, involving 2029 outpatients aged 15-50 years from the Shanghai Mental Health Center. BPD traits were assessed using the Personality Diagnostic Questionnaire 4th Edition Plus (PDQ-4+), and CM experiences were evaluated using the Child Trauma Questionnaire Short Form (CTQ-SF). Participants were categorized into three age groups: adolescents (15-21 years), young adults (22-30 years), and older adults (31-50 years).

**Results:**

Adolescents reported significantly higher frequencies of BPD traits and diagnoses compared to young adults and older adults (*p*=0.036). Specifically, identity disturbance and impulsivity were more pronounced in adolescents (*p*<0.001). Additionally, adolescents reported higher levels of emotional (*F*=15.987, *p*<0.001) and physical abuse (*F*=12.942, *p*=0.002), while older adults reported higher levels of emotional and physical neglect. Logistic regression analysis identified key BPD criteria and CM subtypes that differentiated adolescents from adults.

**Discussion:**

The findings underscore the importance of age-specific interventions in treating BPD and addressing childhood maltreatment. Adolescents exhibit distinct patterns of BPD traits and CM experiences, necessitating tailored therapeutic approaches.

## Introduction

In recent years, the clinical presentation of Borderline Personality Disorder (BPD) among adolescents has become a common diagnosis encountered in psychiatric outpatient clinics (1, 2). The prevalence of behaviors such as non-suicidal self-injury (NSSI) (3, 4) has seen a significant rise in these settings. This increase in BPD and other mental health issues among younger populations highlights the need to consider age-related factors in a broader context, emphasizing the necessity for targeted mental health interventions and policies. However, despite BPD being classified as a Cluster B personality disorder (PD), it is often overlooked in the psychiatric practice in China (5, 6). This oversight has led to significant gaps in the assessment and understanding of the clinical distribution of BPD (7). The paradox of a noticeable increase in clinical prevalence alongside a lack of effective assessment warrants the attention of Chinese psychiatrists. Research has highlighted the importance of early identification and intervention in BPD to mitigate its long-term impact on individuals’ mental health (8, 9). Despite these findings, the diagnostic processes and clinical frameworks in China have not adequately integrated BPD assessments, leading to underdiagnoses and mismanagement of affected adolescents.

The clinical presentation of BPD exhibits significant age-related effects. Naturalistic longitudinal studies of BPD indicate that symptoms improve over time (10). Previous research has indicated that BPD traits are more pronounced in younger individuals, with the severity of symptoms tending to decrease with age (11, 12). Recent research by Michael et al. found that early intervention for BPD is effective across adolescence but manifests differently: it prevents the normative increase of BPD pathology in younger adolescents and significantly decreases BPD pathology in older adolescents (13). The study suggests that developmentally adapted therapeutic interventions could potentially enhance benefits for younger adolescents. This raises an important question within the context of Chinese psychiatric clinical populations: Does the age distribution of BPD follow a similar trend? Furthermore, is this trend consistent across different psychiatric conditions? Exploring these questions is crucial, particularly given that childhood maltreatment (CM) is a well-documented risk factor for BPD. Studies have consistently shown that the more extensive the experience of CM, the higher the risk of developing BPD. Research indicates that different types of CM can uniquely contribute to specific BPD traits, highlighting the importance of addressing these experiences in therapeutic interventions (14–17). However, there is a notable gap in the literature regarding whether the relationship between this risk factor and BPD varies with age.

Building on the aforementioned context, this study aims to investigate and compare self-reported BPD traits, BPD diagnoses, and CM experiences among adolescent, young adult, and older adult patients. Additionally, we intend to conduct stratified analyses across different Axis I diagnoses and genders. Our hypothesis proposes that specific BPD traits and types of CM may demonstrate age-related differences, particularly more pronounced among adolescents within the extensive clinical population.

## Methods

### Participants and study setting

The survey took place at the Shanghai Mental Health Center (SMHC) from 2019 to 2023, targeting outpatients from psycho-counseling and psychiatric clinics at SMHC, one of China’s largest healthcare institutions. The study was approved by the SMHC Research Ethics Committee (2019-17R), and all participants gave written informed consent during recruitment. The objective was to determine the prevalence of PDs in a continuous clinical sample of adult patients. A total of 2029 outpatients were randomly selected between January 2019 and December 2023, based on inclusion criteria such as being aged between 15 and 50 years, capable of understanding the study questionnaire, willing to provide information on PDs and CM, having stable treatment conditions, and having been diagnosed with either psychotic disorders, mood disorders, or anxiety disorders in the outpatient setting. The exclusion criteria included having severe or unstable physical conditions, defined as any medical conditions that could significantly affect a participant’s ability to reliably engage in the study. This includes recent surgeries, uncontrolled chronic illnesses (such as diabetes or heart disease), acute infections, or other health issues that may lead to fluctuating physical or mental states. Additionally, the criteria included being currently pregnant or other factors identified by investigators that would make the patient ineligible.

### BPD assessments

The assessment of BPD traits and symptoms employed a concise, well-structured self-report instrument: The Personality Diagnostic Questionnaire 4^th^ Edition Plus (PDQ-4+) (18), as documented in prior studies (7, 19, 20). The PDQ-4+ consists of 107 true-false questions that evaluate 10 Axis II DSM-IV PDs, with a specific focus on BPD for this study. The questionnaire includes 11 items related to BPD traits, corresponding to the 9 diagnostic criteria in DSM-IV. BPD traits are identified when an individual reports five or more positive criteria, aligning with the DSM-IV requirement of meeting five or more diagnostic criteria for BPD. The primary purpose of the PDQ-4+ is to distinguish individuals exhibiting PD traits from those who do not. The PDQ-4+ has high sensitivity (0.89) and acceptable specificity (0.65), making it a widely used tool for screening DSM-IV PDs among Chinese psychiatric patients. It has demonstrated high test-retest reliability (0.92) in the Chinese population, confirming its consistency in producing reliable results (5, 14, 21).

The nine diagnostic criteria for BPD along with the corresponding items from the PDQ-4+ are as follows: The criterion of “frantic efforts to avoid real or imagined abandonment” (1) is reflected in PDQ-4+ items such as, “To prevent the people I love from leaving me, I would go to extremes” (Item-6) and “Once I realize that someone close to me is no longer getting close to me, I feel very upset and make various strong reactions” (Item-100). Similarly, the criterion of “a pattern of unstable and intense interpersonal relationships” (2) corresponds to items like, “I either like or admire someone, or I resent them, without any feelings in between” (Item-19) and “My relationships with others sometimes become very intimate, and sometimes full of resentment” (Item-101). Other criteria, such as “identity disturbance” (3), “impulsivity in at least two areas that are potentially self-damaging” (4), and “recurrent suicidal behavior, gestures, or threats, or self-mutilating behavior” (5) are linked to specific items in the PDQ-4+, like “I often want to figure out who I really am” (Item-32) and “I have tried to hurt myself or commit suicide” (Item-45). Additionally, “affective instability due to a marked reactivity of mood” (6) is represented by “I am a person with unstable emotions” (Item-58), while “chronic feelings of emptiness” (7) corresponds to “I feel that my life is dull and meaningless” (Item-69). The criterion of “inappropriate, intense anger or difficulty controlling anger” (8) is reflected in “I have difficulty controlling my anger or temper” (Item-78), and finally, “transient, stress-related paranoid ideation or severe dissociative symptoms” (9) is illustrated by “When faced with stressful situations, I become sensitive, suspicious, or forgetful about things I just did” (Item-93).

The diagnosis of BPD in this study was conducted using the Structured Clinical Interview for DSM-IV Axis II (SCID-II), a semi-structured clinical interview designed for diagnosing personality disorders based on DSM-IV criteria. Our team translated and implemented the Chinese version of the SCID-II. The results obtained with SCID-II show high consistency (0.90) with clinical diagnoses, and the test-retest reliability is satisfactory (0.70) (22). The SCID-II assessments were carried out by trained research personnel with a minimum of two years of professional experience and specific training in administering the SCID-II. This study primarily focused on cases that met the diagnostic criteria for BPD as assessed by the SCID-II.

### CMs assessment

CM was evaluated using the Chinese version of the Child Trauma Questionnaire Short Form (CTQ-SF) (16, 23, 24). This questionnaire includes 28 self-report items divided into five subscales: emotional abuse (EA), physical abuse (PA), sexual abuse (SA), emotional neglect (EN), and physical neglect (PN). Participants rated each item’s frequency on a 5-point scale, ranging from 1 (never) to 5 (always), with higher scores reflecting greater maltreatment. The Chinese CTQ-SF has been validated as a reliable tool for assessing CM in Chinese clinical populations (15–17). A participant was considered to have experienced CM if they scored (i) 8 or above on the PA, SA, or PN subscales; (ii) 10 or above on the EA subscale; and/or (iii) 15 or above on the EN subscale.

### Statistical analyses

Statistical analyses were conducted using SPSS for Windows (version 20.0), with statistical significance set at *p*<0.05. Quantitative variables are presented as mean ± standard deviation (SD), while qualitative variables are expressed as frequencies (%). Participants were categorized into three age groups: Adolescents (15-21 years) (25, 26), Young Adults (22-30 years), and Older Adults (31-50 years). We defined adolescents as ages 15-21 and young adults as ages 22-30 based on developmental psychology, which recognizes significant cognitive and emotional changes during these transitional periods. Specifically, the age of 21 marks the end of adolescence (27), aligning with the onset of emerging adulthood, while 30 is often considered a milestone for young adulthood, where individuals typically experience increased stability in various life domains. The study compared the frequencies of BPD traits and diagnoses across these age groups. Additionally, frequencies of BPD criteria were analyzed across different age groups stratified by diagnoses of psychotic disorders, mood disorders, and anxiety disorders. Radar charts were used to illustrate comparisons of various CM characteristics among males, females, individuals without BPD traits, and those with BPD traits across the different age groups. Self-reported CM characteristics and frequencies were also compared, stratified by the presence or absence of BPD traits among Adolescents, Young Adults, and Older Adults. Pearson correlation coefficients were calculated to assess the relationships between CM characteristics and total score of BPD traits across different age groups. To identify factors associated with age groups, a logistic regression model was employed. The model included the 9 BPD criteria and 5 CM subtypes. The results were reported with β coefficients, 95% confidence intervals (CI), and P-values from Wald tests. This analysis aims to comprehensively examine the relationship between age and a range of variables, rather than focusing on individual factors.

## Results

The sociodemographic and clinical information for the 2029 participants, divided into three age groups, is detailed in **Table 1**. Participants ranged in age from 15 to 50 years, with an average age of 26.80 ± 8.718 years. The sample included 674 adolescents (33.2%), 743 young adults (36.6%), and 612 older adults (30.2%). Among older adults, there was a higher percentage of women, married individuals, and those with more than 10 visits compared to adolescents and young adults. Additionally, older adults had a longer duration of psychiatric disorders. In contrast, the percentage of participants with college or higher education was greater among young adults than in the other two groups.

**Table 1:** Socio-demographic characteristics of the overall sample: a comparison among adolescents, young adults, and older adults.

	Adolescents		Young Adults		Older Adults		*χ^2^*	*p*
	N/Means	%/SD.	N/Means	%/SD.	N/Means	%/SD.		
Cases	674		743		612		–	–
Age	18.17	1.972	25.39	2.570	38.04	5.378	1801.398	**<0.001**
Sex (Man)	329	48.8%	342	46.0%	229	37.4%	18.203	**<0.001**
Sex (Woman)	345	51.2%	401	54.0%	383	62.6%		
Education								
Middle or high school	377	55.9%	338	45.5%	383	62.6%	40.820	**<0.001**
College or higher	297	44.1%	405	54.5%	229	37.4%		
Marriage								
Single	648	96.1%	450	60.6%	174	28.4%	631.004	**<0.001**
Married	26	3.9%	293	39.4%	438	71.6%		
Self-reported Pre-illness characteristic								
Introversion	296	43.9%	298	40.1%	249	40.7%	4.126	0.389
Middle type	271	40.2%	314	42.3%	251	41.0%		
Extroversion	107	15.9%	131	17.6%	112	18.3%		
Family history of mental disorder								
With family history	60	8.9%	90	12.1%	80	13.1%	6.254	**0.044**
Without family history	614	91.1%	653	87.9%	532	86.9%		
Visits and Course								
First Visit	388	57.6%	419	56.4%	324	52.9%	78.431	**<0.001**
2-10 visits	208	30.8%	204	27.4%	111	18.1%		
>10 visits	78	11.6%	120	16.2%	177	28.9%		
Course of illness (months)	27.34	29.421	46.37	48.989	90.41	95.291	110.541	**<0.001**
Diagnostic Category								
Psychotic disorders	198	29.4%	208	28.0%	174	28.4%	0.941	0.919
Mood disorders	197	29.2%	234	31.5%	189	30.9%		
Anxiety disorders	279	41.4%	301	40.5%	249	40.7%		

*χ^2^* for kappa test or Kruskal-Wallis test. Significant values are indicated in bold.

**Table 2** presents the frequencies of BPD traits and diagnosis across adolescents, young adults, and older adults. The data reveal significant differences in several BPD criteria among the age groups. For instance, Criteria 3 and Criteria 4 show significantly higher frequencies in adolescents compared to the other groups, with p-values <0.001. The overall prevalence of individuals meeting the threshold for BPD traits (Criteria >=5) is also significantly higher in adolescents and young adults compared to older adults, with a p-value of 0.036. Additionally, the structured interview results indicate a significantly higher BPD diagnosis rate among adolescents and young adults than older adults, with a p-value <0.001.

**Table 2:** Frequencies of borderline personality disorder (BPD) traits and diagnosis: a comparison among adolescents, young adults, and older adults.

PDs	Adolescents		Young Adults		Older Adults		*χ^2^*	*p*
	N	%	N	%	N	%		
Self-reported BPD traits								
Criteria 1	400	59.3%	482	64.9%	377	61.6%	4.656	0.097
Criteria 2	402	59.6%	415	55.9%	334	54.6%	3.721	0.156
Criteria 3	412	61.1%	410	55.2%	308	50.3%	15.288	**<0.001**
Criteria 4	286	42.4%	367	49.4%	235	38.4%	17.214	**<0.001**
Criteria 5	272	40.4%	269	36.2%	215	35.1%	4.305	0.116
Criteria 6	533	79.1%	573	77.1%	464	75.8%	1.996	0.369
Criteria 7	359	53.3%	404	54.4%	302	49.3%	3.645	0.162
Criteria 8	385	57.1%	442	59.5%	384	62.7%	4.234	0.120
Criteria 9	504	74.8%	546	73.5%	436	71.2%	2.082	0.353
BPD trait (Criteria >=5)	403	59.8%	448	60.3%	330	53.9%	6.649	**0.036**
Structured interview BPD								
BPD diagnosis	71	10.5%	86	11.6%	21	3.4%	31.718	**<0.001**

*χ^2^* for kappa test. Diagnostic Criteria: 1. Frantic efforts to avoid real or imagined abandonment; 2. A pattern of unstable and intense interpersonal relationships; 3. Identity disturbance; 4. Impulsivity in at least two areas that are potentially self-damaging; 5. Recurrent suicidal behavior, gestures, or threats, or self-mutilating behavior; 6. Affective instability due to a marked reactivity of mood; 7. Chronic feelings of emptiness; 8. Inappropriate, intense anger or difficulty controlling anger; 9. Transient, stress-related paranoid ideation or severe dissociative symptoms. BPD traits are defined as meeting at least 5 out of the 9 criteria for Borderline Personality Disorder (BPD) on self-reported questionnaires. In contrast, a BPD diagnosis is established through a structured face-to-face interview. After applying the Bonferroni correction for 9 comparisons, a p-value of less than approximately 0.0056 is considered statistically significant. Significant values are indicated in bold.

**Figure 1:**
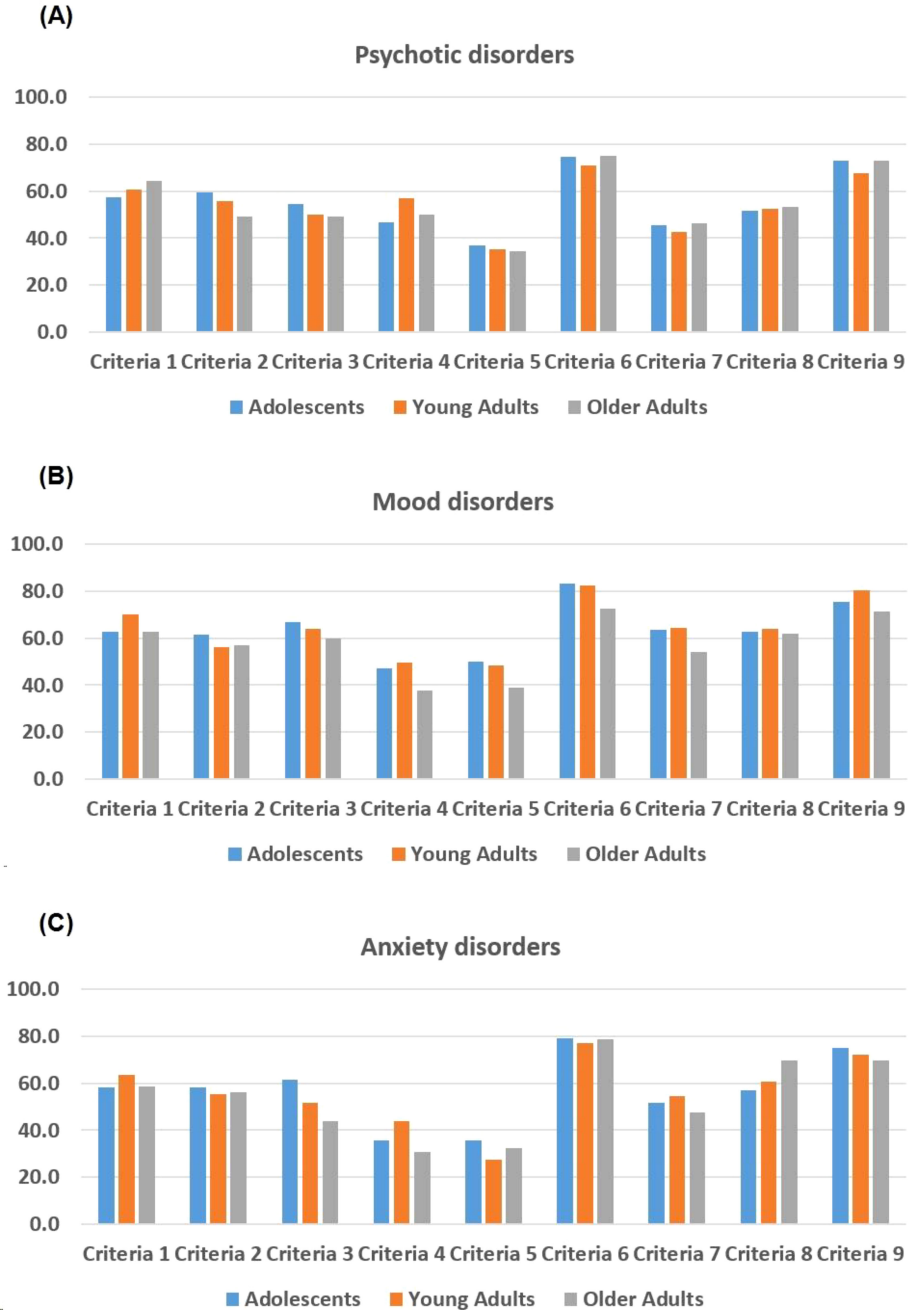
Frequencies of Borderline Personality Disorder (BPD) criteria across different age groups and psychiatric disorders **(A)** Psychotic disorders; **(B)** Mood disorders; **(C)** Anxiety disorder. Diagnostic Criteria: 1. Frantic efforts to avoid real or imagined abandonment; 2. A pattern of unstable and intense interpersonal relationships; 3. Identity disturbance; 4. Impulsivity in at least two areas that are potentially self-damaging; 5. Recurrent suicidal behavior, gestures, or threats, or self-mutilating behavior; 6. Affective instability due to a marked reactivity of mood; 7. Chronic feelings of emptiness; 8. Inappropriate, intense anger or difficulty controlling anger; 9. Transient, stress-related paranoid ideation or severe dissociative symptoms.

**Figure 1** illustrates the distribution of BPD criteria across three age groups—adolescents, young adults, and older adults—within different psychiatric conditions: psychotic disorders (**Figure 1A**), mood disorders (**Figure 1B**), and anxiety disorders (**Figure 1C**). In psychotic disorders (**Figure 1A**), adolescents exhibit higher frequencies for Criteria 1, 3, 4, 6, and 9 compared to young and older adults. Young adults display higher frequencies for Criteria 5 and 7, while older adults have relatively lower frequencies across most criteria. For mood disorders (**Figure 1B**), adolescents also show higher frequencies for Criteria 1, 4, 6, and 9. Young adults have the highest frequencies for Criteria 2 and 3, whereas older adults have slightly higher frequencies for Criteria 5 and 7. Regarding anxiety disorders (**Figure 1C**), adolescents again lead in frequencies for Criteria 1, 3, 4, 6, and 9. Young adults present higher frequencies for Criteria 2 and 5, while older adults show higher frequencies for Criteria 7 and 8. Overall, adolescents tend to exhibit higher frequencies for several BPD criteria across all three psychiatric conditions, particularly for Criteria 1, 4, 6, and 9, indicating a pronounced presence of these traits within this age group.

As shown in **Table 3**, in patients with BPD traits (Criteria >= 5), the mean scores for EA and PA were significantly higher across all age groups, with adolescents reporting the highest mean scores (EA: 8.70, PA: 6.75). Significant differences were observed in the frequencies of EA and PA, with the highest percentages in adolescents (EA: 33.0%, PA: 24.6%). In contrast, the absence of BPD traits (Criteria < 5) also showed significant differences in EA and PA scores among age groups, with adolescents reporting higher mean scores (EA: 7.83, PA: 6.44).

**Table 3:** Self-reported childhood maltreatment (CM) characteristics and frequencies stratified by presence or absence of borderline personality disorder (BPD) traits: a comparison among adolescents, young adults, and older adults.

CMs	Adolescents		Young Adults		Older Adults		*F*/*χ^2^*	*p*
	N/Means	%/SD.	N/Means	%/SD.	N/Means	%/SD.		
Presence of BPD Trait (Criteria >= 5) (N=1181)								
Score [Mean (SD)]								
EA	8.70	3.826	8.24	3.580	7.58	3.101	15.987	**<0.001**
PA	6.75	2.796	6.54	2.583	6.22	2.412	12.942	**0.002**
SA	6.13	2.242	6.09	2.245	5.99	2.047	0.607	0.738
EN	12.83	5.145	12.87	5.006	13.47	4.631	5.714	0.057
PN	9.10	3.156	9.09	3.310	9.42	2.961	5.213	0.074
Frequency [N(%)]								
EA	133	33.0%	126	28.1%	66	20.0%	15.511	**<0.001**
PA	99	24.6%	97	21.7%	51	15.5%	9.343	**0.009**
SA	61	15.1%	74	16.5%	44	13.3%	1.499	0.473
EN	142	35.2%	161	35.9%	138	41.8%	3.968	0.138
PN	271	67.2%	285	63.6%	236	71.5%	5.377	0.068
Absence of BPD Trait (Criteria < 5) (N=848)								
Score [Mean (SD)]								
EA	7.83	3.030	7.10	2.428	6.80	2.513	21.498	**<0.001**
PA	6.44	2.439	6.02	1.958	5.91	2.075	14.492	**0.001**
SA	5.85	1.773	5.77	1.759	5.70	1.434	1.196	0.550
EN	11.90	4.782	11.58	4.368	12.78	4.646	10.298	**0.006**
PN	8.85	2.945	8.67	3.055	9.11	2.855	4.555	0.103
Frequency [N(%)]								
EA	61	22.5%	45	15.3%	31	11.0%	13.802	**0.001**
PA	58	21.4%	40	13.6%	35	12.4%	9.992	**0.007**
SA	31	11.4%	31	10.5%	30	10.6%	0.146	0.930
EN	76	28.0%	70	23.7%	93	33.0%	6.098	0.047
PN	170	62.7%	166	56.3%	186	6.0%	5.948	0.051

*F* values for one-way ANOVA test, *χ^2^* for kappa test. Abbreviations: EA, Emotional abuse; PA, Physical abuse; SA, Sexual abuse; EM, Emotional neglect; PN, Physical neglect. After applying the Bonferroni correction for 5 comparisons, a p-value of less than approximately 0.01 is considered statistically significant. Significant values are indicated in bold.

**Table 4** presents the correlations between CM characteristics and BPD traits among adolescents, young adults, and older adults. For adolescents, EA showed a statistically significant positive correlation with the total score of BPD traits (*r* = 0.182, *p* < 0.001). PA and SA also demonstrated significant correlations (PA: *r* = 0.102, *p* = 0.008; SA: *r* = 0.092, *p* = 0.017). EN and PN showed weaker correlations, with EN showing a significant correlation (*r* = 0.109, *p* = 0.005), while PN did not reach significance after correction (*r* = 0.083, *p* = 0.032). Among young adults, the patterns were similar, with EA (*r* = 0.267, *p* < 0.001), PA (*r* = 0.166, *p* < 0.001), and SA (*r* = 0.152, *p* < 0.001) all exhibiting significant positive correlations with BPD traits. EN also showed a significant correlation (*r* = 0.177, *p* < 0.001), while PN (*r* = 0.089, *p* = 0.015) was notable but less pronounced. In older adults, EA remained significant (*r* = 0.117, *p* = 0.004), but the correlations for PA (*r* = 0.068, *p* = 0.095), SA (*r* = 0.087, *p* = 0.032), EN (*r* = 0.036, *p* = 0.377), and PN (*r* = 0.030, *p* = 0.458) were weaker and not statistically significant. The correlations between CM characteristics among adolescents, young adults, and older adults are detailed in the **Supplementary Table S1**.

**Table 4:** Correlations between Self-reported Childhood Maltreatment (CM) Characteristics and Borderline Personality Disorder (BPD) Traits Among Adolescents, Young Adults, and Older Adults.

CMs	Adolescents		Young Adults		Older Adults	
	*r*	*p*	*r*	*p*	*r*	*p*
	Total score of BPD traits					
EA	**0.182**	**<0.001**	**0.267**	**<0.001**	**0.117**	**0.004**
PA	**0.102**	**0.008**	**0.166**	**<0.001**	0.068	0.095
SA	0.092	0.017	**0.152**	**<0.001**	0.087	0.032
EN	**0.109**	**0.005**	**0.177**	**<0.001**	0.036	0.377
PN	0.083	0.032	0.089	0.015	0.030	0.458

*r* values for Pearson Correlation. Abbreviations: EA, Emotional abuse; PA, Physical abuse; SA, Sexual abuse; EM, Emotional neglect; PN, Physical neglect. After applying the Bonferroni correction for 5 comparisons, a p-value of less than approximately 0.01 is considered statistically significant. Significant values are indicated in bold.

**Figure 2:**
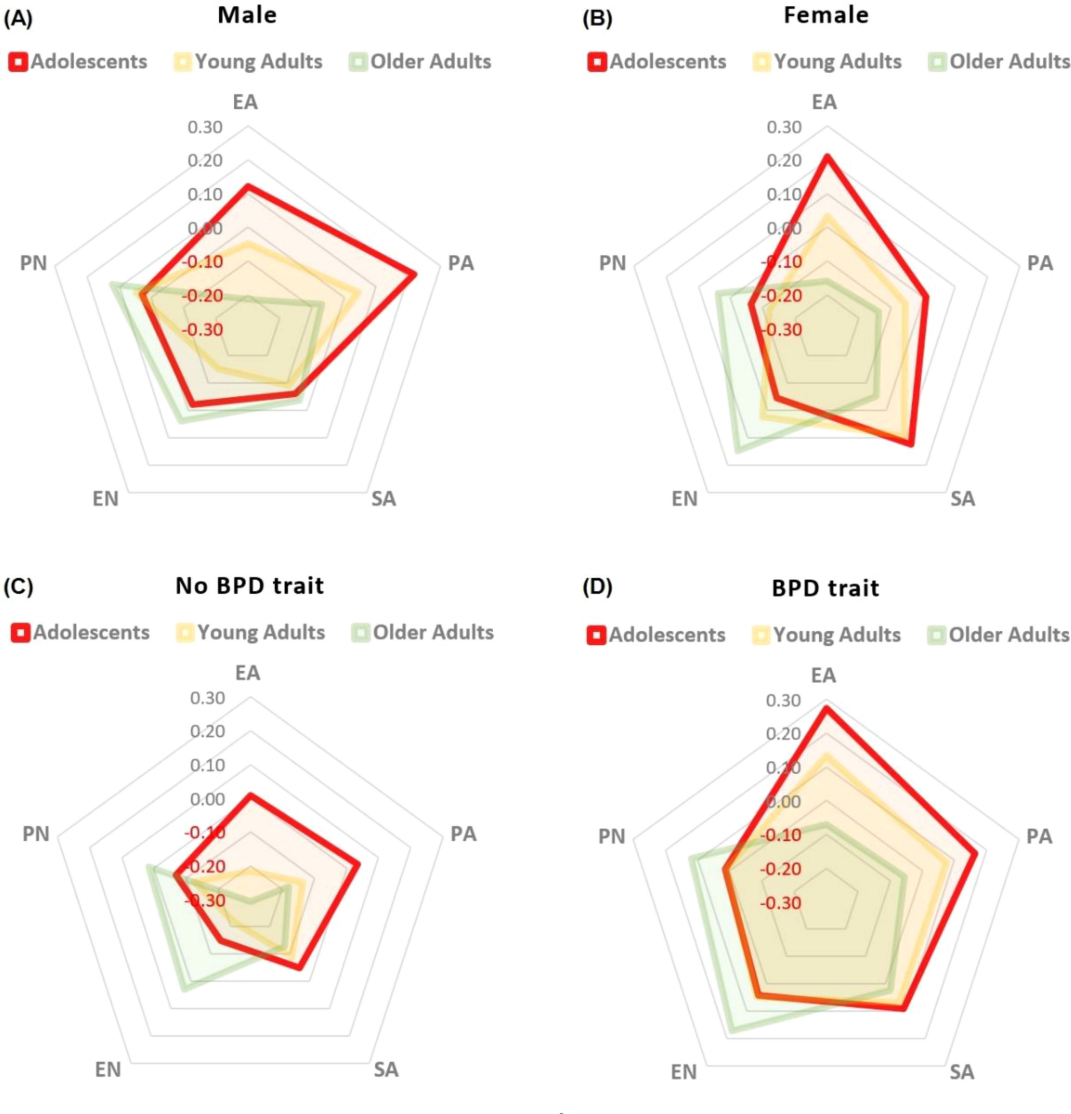
Comparative analysis of self-reported Childhood Maltreatment (CM) Characteristics Across Age Groups in Males **(A)**, Females **(B)**, and Individuals without **(C)** or with **(D)** Borderline Personality Disorder (BPD) Trait. EA, Emotional abuse; PA, Physical abuse; SA, Sexual abuse; EM, Emotional neglect; PN, Physical neglect.

**Figure 2** presents radar charts illustrating the comparison of different age groups (adolescents, young adults, and older adults) across various CMs for males (**Figure 2A**), females (**Figure 2B**), individuals without BPD traits (**Figure 2C**), and individuals with BPD traits (**Figure 2D**). Across all subgroups, adolescents consistently exhibit higher scores in the evaluated dimensions compared to young adults and older adults in EA and PA, while older adults consistently exhibit higher scores in EN and PN for both genders and regardless of BPD trait presence. In male patients, age differences in PA scores are more pronounced than in females, while among patients without BPD traits, age differences in EA, PA, and EN are more noticeable compared to those with BPD traits. Additionally, patients with BPD traits report higher scores across CMs in all age groups compared to patients without BPD traits.

The logistic regression analysis presented in **Table 5** demonstrates significant predictors for differentiating between adolescents and adults based on BPD criteria and CM. Specifically, Criteria 1 (*β* = 1.352, *p* = 0.005), Criteria 3 (*β* = 0.703, *p* = 0.001), and Criteria 8 (*β* = 1.368, *p* = 0.005) were significantly associated with distinguishing the age groups. Among the CM variables, EA was a significant predictor (*β* = 1.097, *p* < 0.001), indicating that higher EA scores increase the likelihood of being classified as an adolescent. Conversely, EN showed a significant negative association (*β* = 0.966, *p* = 0.003), suggesting that higher EN scores decrease the likelihood of being classified as an adolescent.

**Table 5:** Logistic regression for differentiating adolescents and adults by Borderline Personality Disorder (BPD) criteria and Childhood Maltreatment (CM).

Variables	Analysis						
	Beta	S.E.	*β*	95%CI for *β*		*Wald*	*P*
Criteria 1 (0: No, 1: Yes)	0.301	0.108	1.352	1.095	1.669	7.847	**0.005**
Criteria 2 (0: No, 1: Yes)	-0.192	0.106	0.826	0.670	1.016	3.261	0.071
Criteria 3 (0: No, 1: Yes)	-0.353	0.102	0.703	0.575	0.858	11.933	**0.001**
Criteria 4 (0: No, 1: Yes)	0.189	0.104	1.208	0.985	1.480	3.302	0.069
Criteria 5 (0: No, 1: Yes)	-0.188	0.108	0.829	0.670	1.025	3.001	0.083
Criteria 6 (0: No, 1: Yes)	-0.167	0.133	0.846	0.652	1.099	1.569	0.210
Criteria 7 (0: No, 1: Yes)	0.093	0.104	1.098	0.896	1.346	0.810	0.368
Criteria 8 (0: No, 1: Yes)	0.313	0.110	1.368	1.102	1.698	8.063	**0.005**
Criteria 9 (0: No, 1: Yes)	-0.106	0.118	.900	0.713	1.135	0.799	0.371
EA	0.093	0.019	1.097	1.058	1.138	24.630	**<0.001**
PA	0.032	0.023	1.033	0.988	1.080	2.039	0.153
SA	-0.024	0.026	0.976	0.928	1.027	0.862	0.353
EN	-0.035	0.012	0.966	0.944	0.988	8.618	**0.003**
PN	-0.026	0.018	0.974	0.940	1.009	2.115	0.146
Constant	-0.803	0.247	0.448			10.594	**0.001**

Beta is the regression coefficient. S.E. is the standard error. 95% CI is the estimated 95% confidence interval for the corresponding parameter. β is the standardized regression coefficient. Abbreviations: Diagnostic Criteria: 1. Frantic efforts to avoid real or imagined abandonment; 2. A pattern of unstable and intense interpersonal relationships; 3. Identity disturbance; 4. Impulsivity in at least two areas that are potentially self-damaging; 5. Recurrent suicidal behavior, gestures, or threats, or self-mutilating behavior; 6. Affective instability due to a marked reactivity of mood; 7. Chronic feelings of emptiness; 8. Inappropriate, intense anger or difficulty controlling anger; 9. Transient, stress-related paranoid ideation or severe dissociative symptoms. EA, Emotional abuse; PA, Physical abuse; SA, Sexual abuse; EM, Emotional neglect; PN, Physical neglect. Significant values are indicated in bold.

## Discussion

### Key findings

This study reveals significant age-related differences in the frequency and characteristics of BPD traits and diagnoses, as well as CM experiences among adolescents, young adults, and older adults. Adolescents exhibit higher frequencies of several BPD criteria, particularly Criteria 3 and 4, with a significantly greater overall prevalence of BPD traits compared to older adults. BPD diagnosis rates are notably higher among adolescents and young adults. Analysis of psychiatric conditions shows that adolescents consistently display higher frequencies of certain BPD criteria across psychotic, mood, and anxiety disorders. CM experiences, specifically EA and PA, are reported more frequently and with higher severity by adolescents, especially those with BPD traits. Logistic regression identifies Criteria 1, 3, and 8, along with EA, as significant predictors for differentiating between adolescents and adults, while EN is more commonly associated with older adults. These findings underscore the pronounced presence of BPD traits and specific CM experiences in adolescents within the clinical population.

### BPD traits in adolescents

Our analysis revealed significant differences in the distribution of BPD criteria among adolescents compared to young and older adults, particularly in the context of various psychiatric conditions. Adolescents displayed elevated frequencies of Criteria 1 (frantic efforts to avoid abandonment), 3 (identity disturbance), 4 (impulsivity), 6 (affective instability), and 9 (paranoid ideation or dissociative symptoms). This prevalence may reflect developmental vulnerabilities inherent in adolescence, a period characterized by identity exploration and emotional reactivity. Consistent with a substantial body of research (10, 11, 28), this study found that BPD traits are more pronounced in adolescents compared to adults, with BPD diagnoses being more common among the younger population (29). Several factors might contribute to this age effect (30). First, adolescence is a critical period for emotional and psychological development, characterized by heightened emotional sensitivity and instability (31). Second, adolescents often face significant life changes and stressors, such as peer pressure and academic challenges, which can exacerbate BPD symptoms. Third, the ongoing development of the prefrontal cortex, which is crucial for impulse control and emotional regulation, might make adolescents more vulnerable to the impulsive and unstable behaviors associated with BPD (32, 33).

Further analysis of BPD criteria revealed that Criteria 3 (Identity disturbance) and Criteria 4 (Impulsivity in at least two areas that are potentially self-damaging) are particularly prominent in adolescents. This may be attributed to several factors related to the unique characteristics of adolescent psychological development (1). First, identity formation is a central task during adolescence, and the instability in self-image and identity observed in BPD may reflect this normative developmental challenge (34). Second, the impulsivity noted in adolescents with BPD can be linked to the developmental immaturity of brain regions involved in self-regulation and decision-making, which are still maturing during adolescence (35). Third, adolescents are more likely to engage in risk-taking behaviors as part of exploring their autonomy and identity, which can manifest as impulsivity in those with BPD traits. These developmental dynamics underscore the heightened vulnerability of adolescents to BPD traits and highlight the importance of early intervention and targeted therapeutic strategies (36).

### CM in adolescents

It is unsurprising that adolescents report experiencing CM more frequently than adults (37), particularly in abuse-related categories, whereas adults are more likely to report neglect-related CM. Several factors could explain this discrepancy: First, adolescents may have a heightened awareness and immediate recall of recent experiences of abuse. Given their proximity to these events, they might perceive and report abuse more acutely compared to adults, who might have more time-distanced and less vivid memories of their childhood experiences (38, 39). Second, adolescents might be more likely to recognize and label certain behaviors as abusive due to increased societal awareness and education about abuse. In contrast, adults might have grown up in environments where certain abusive behaviors were normalized and, therefore, may not identify them as abuse when reflecting back. Third, the social environment has evolved, with greater awareness and less tolerance of abusive behaviors in recent years. Adolescents might be more encouraged and supported in reporting abuse due to these societal changes, which were less prevalent during the childhood of the older adults (40).

### Correlation Between BPD Traits and CM

The findings indicate that the correlation between CM types and BPD traits varies significantly across age groups. Notably, the relationships observed in the adolescent and young adult groups were stronger compared to the older adult group. This trend may be attributed to two potential factors. First, as individuals age, there may be a natural decrease in BPD traits (12) and the influence of CM, as individuals develop coping mechanisms and emotional regulation strategies over time. Second, older adults might experience retrospective recall biases, leading to underreporting or diminished recollection of childhood maltreatment experiences, which could weaken the observed correlations. Furthermore, the stronger association of abuse types of CM—such as emotional, physical, and sexual abuse—with BPD traits underscores the profound impact these early adverse experiences can have on personality development. Abuse often instills profound feelings of insecurity and affects interpersonal relationships, which are core components of BPD. This highlights the critical importance of addressing these specific types of maltreatment in therapeutic interventions, as they may play a pivotal role in the manifestation and persistence of BPD traits throughout an individual’s life.

### Clinical relevance

The key findings of this study hold significant clinical implications for the psychological treatment of BPD and CM across different age groups. Firstly, the pronounced presence of BPD traits and higher rates of BPD diagnosis in adolescents suggest a critical need for early identification and intervention in this demographic. Early intervention strategies could potentially mitigate the progression of BPD symptoms and improve long-term outcomes. Research indicates that adolescents who receive timely and appropriate treatment for BPD exhibit better psychological functioning and reduced symptom severity over time (41–43). Secondly, the age-specific patterns of CM experiences, with adolescents reporting more abuse-related CM and adults reporting more neglect-related CM, highlight the necessity for tailored therapeutic approaches. For adolescents, interventions might need to focus more on addressing the immediate psychological impacts of abuse, utilizing trauma-focused therapies that incorporate components of emotion regulation and interpersonal effectiveness. In contrast, for adults, therapy might benefit from a greater emphasis on addressing long-term issues associated with neglect, such as chronic feelings of emptiness and relationship difficulties.

Lastly, the differential impact of specific BPD criteria, such as identity disturbance and impulsivity being more pronounced in adolescents, underscores the importance of developmentally appropriate interventions. Adolescents are at a crucial stage of identity formation, and impulsive behaviors can have far-reaching consequences. Therapeutic approaches that support identity development and provide skills to manage impulsivity could be particularly beneficial. Dialectical Behavior Therapy (DBT) and Cognitive Behavioral Therapy (CBT) have been shown to be effective in addressing these aspects in young populations (44, 45). The identified age-related differences in BPD traits and CM experiences suggest that psychological interventions should be tailored to address these specific developmental contexts. For younger individuals, therapies may focus on building emotional regulation skills and addressing attachment issues stemming from early maltreatment, whereas for older individuals, interventions might emphasize coping strategies and resilience-building based on their accumulated life experiences. In conclusion, the findings from this study emphasize the importance of age-specific, tailored interventions for BPD and CM. Early and targeted therapeutic strategies can significantly improve the prognosis for adolescents and adults with BPD, addressing both the immediate and long-term psychological effects of childhood maltreatment. This approach not only enhances clinical outcomes but also contributes to the overall mental health and well-being of affected individuals.

### Limitations

Several limitations should be considered when interpreting the findings of this study. Firstly, the cross-sectional design restricts our ability to infer causality. While the study offers valuable descriptive comparisons, future research employing longitudinal designs, such as cohort studies, would be more effective in uncovering causal relationships. Secondly, recall bias is an inherent issue in retrospective assessments. The accuracy of participants’ recollections may be influenced by their varying illness trajectories and ages. Psychiatric symptoms such as depression, anxiety, and psychosis could further distort the recollection of childhood experiences, potentially leading to inaccuracies, exaggerations of neglect, or the fabrication or misconstruction of abusive events. Thirdly, the measurement of BPD traits with only one or two items per trait may lead to unstable estimates, and the significant correlations between traits could affect the interpretation of the regression analysis. However, the inclusion of a relatively large sample size in this study may partially mitigate these issues by enhancing the reliability of the findings. Fourthly, using a specific age cut-off to categorize age groups may not be entirely suitable, as it can overlook important developmental nuances. Future research could benefit from treating age as a continuous variable and employing quantitative regression analysis methods to enhance the applicability and interpretability of the findings. Lastly, due to the study’s primary focus on age differences of BPD and CM, it did not systematically assess the clinical symptoms of patients with different psychiatric conditions. Consequently, the relationship between various symptom types and BPD and CM remains unexplored. Future studies should aim to incorporate a more comprehensive evaluation of clinical symptoms across different conditions to better understand these relationships.

## Conclusion

This study highlights significant age-related differences in the prevalence of BPD traits, diagnoses, and CM experiences among adolescents, young adults, and older adults. Adolescents reported higher frequencies of BPD traits and diagnoses, particularly for identity disturbance and impulsivity. Childhood maltreatment was also more frequently reported by adolescents, especially in the form of abuse, while adults reported more neglect. These findings underscore the importance of age-specific interventions and highlight the need for longitudinal research to further understand the developmental trajectory and causal mechanisms underlying these differences.

## Data Availability

The original contributions presented in the study are included in the article/**Supplementary Material**. Further inquiries can be directed to the corresponding authors.
