# Supplementary material for: Age-related differences in borderline personality disorder traits and childhood maltreatment: a cross-sectional study
Source: Front Psychiatry. 2025 Jan 22;16:1454328. doi: 10.3389/fpsyt.2025.1454328 (PMC11794515; doi:10.3389/fpsyt.2025.1454328)
Supplement: Supplementary file 1 [file Table1.docx]

**Supplementary**

**Age-Related Differences in Borderline Personality Disorder Traits and Childhood Maltreatment: A Cross-Sectional Study**

Table S1 presents the correlations between self-reported childhood maltreatment (CM) characteristics among adolescents, young adults, and older adults. For adolescents, significant positive correlations were observed between emotional abuse (EA) and physical abuse (PA) (*r* = 0.544, *p* < 0.001), as well as between EA and sexual abuse (SA) (*r* = 0.330, *p* < 0.001). Additionally, EA showed strong correlations with emotional neglect (EN) (*r* = 0.457, *p* < 0.001) and physical neglect (PN) (*r* = 0.400, *p* < 0.001). In young adults, EA also exhibited significant positive correlations with PA (*r* = 0.539, *p* < 0.001), SA (*r* = 0.355, *p* < 0.001), EN (*r* = 0.408, *p* < 0.001), and PN (*r* = 0.333, *p* < 0.001).Among older adults, EA continued to show significant correlations with PA (*r* = 0.458, *p* < 0.001), SA (*r* = 0.356, *p* < 0.001), EN (*r* = 0.301, *p* < 0.001), and PN (*r* = 0.295, *p* < 0.001). Furthermore, PA demonstrated significant correlations with both SA (*r* = 0.298, *p* < 0.001) and EN (*r* = 0.199, *p* < 0.001) in older adults.

**Table s1.** Correlations between Self-reported Childhood Maltreatment (CM) Characteristics Among Adolescents, Young Adults, and Older Adults.

| CMs | EA | | | PA | | | SA | | EN | | PN | |
| --- | --- | --- | --- | --- | --- | --- | --- | --- | --- | --- | --- | --- |
|  | *r* | *p* | *r* | | *p* | *r* | | *p* | *r* | *p* | *r* | *p* |
| Adolescents | | | | | | | | | | | | |
| EA | **-** | **-** | 0.544 | | <0.001 | 0.330 | | <0.001 | 0.457 | <0.001 | 0.400 | <0.001 |
| PA | **-** | **-** | **-** | | **-** | 0.223 | | <0.001 | 0.314 | <0.001 | 0.316 | <0.001 |
| SA | **-** | **-** | **-** | | **-** | **-** | | **-** | 0.103 | 0.007 | 0.225 | <0.001 |
| EN | **-** | **-** | **-** | | **-** | **-** | | **-** | **-** | **-** | 0.498 | <0.001 |
| PN | **-** | **-** | **-** | | **-** | **-** | | **-** | **-** | **-** | **-** | **-** |
| Young Adults | | | | | | | | | | | | |
| EA | **-** | **-** | 0.539 | | <0.001 | 0.355 | | <0.001 | 0.408 | <0.001 | 0.333 | <0.001 |
| PA | **-** | **-** | **-** | | **-** | 0.267 | | <0.001 | 0.272 | <0.001 | 0.262 | <0.001 |
| SA | **-** | **-** | **-** | | **-** | **-** | | **-** | 0.093 | 0.012 | 0.200 | <0.001 |
| EN | **-** | **-** | **-** | | **-** | **-** | | **-** | **-** | **-** | 0.462 | <0.001 |
| PN | **-** | **-** | **-** | | **-** | **-** | | **-** | **-** | **-** | **-** | **-** |
| Older Adults | | | | | | | | | | | | |
| EA | **-** | **-** | 0.458 | | <0.001 | 0.356 | | <0.001 | 0.301 | <0.001 | 0.295 | <0.001 |
| PA | **-** | **-** | **-** | | **-** | 0.298 | | <0.001 | 0.199 | <0.001 | 0.242 | <0.001 |
| SA | **-** | **-** | **-** | | **-** | **-** | | **-** | 0.079 | 0.049 | 0.187 | <0.001 |
| EN | **-** | **-** | **-** | | **-** | **-** | | **-** | **-** | **-** | 0.370 | <0.001 |
| PN | **-** | **-** | **-** | | **-** | **-** | | **-** | **-** | **-** | **-** | **-** |

Note. *r* values for Pearson Correlation. Abbreviations: EA, Emotional abuse; PA, Physical abuse; SA, Sexual abuse; EM, Emotional neglect; PN, Physical neglect.
